# Sibling position and its association with the well-being of mothers

**DOI:** 10.1192/j.eurpsy.2026.11934

**Published:** 2026-07-31

**Authors:** E. Polonetskaya, E. Pervichko

**Affiliations:** Faculty of Psychology, Lomonosov Moscow State University, Moscow, Russian Federation

## Abstract

**Introduction:**

It is commonly known that one’s sibling position may have an influence on their psychological well-being, and, for older siblings, such influence in certain cases may be somewhat negative. This present work continues the line of similar studies focusing on mothers with small children.

**Objectives:**

Our research was aimed at studying the influence of a mother’s sibling position, together with certain other factors, on the basic markers of her psychological well-being such as burnout, depressive tendencies, anxiety and optimism, during the first years of motherhood.

**Methods:**

A maternal burnout questionnaire in Russian (the Maternal Burnout Assessment Tool, MBAT), being an adaptation of the Burnout Assessment Tool to the motherhood context, was used to assess burnout tendencies in mothers. To measure optimism, depression tendencies and anxiety, the LOT-R, BDI and State-Trait Anxiety Inventory by C. Spielberger in their respective Russian adaptations were used. The surveys were conducted between September 2023 and January 2025. The sample numbers are from 356 to 792 Russian speaking mothers (as shown below) with children up to seven years of age. Single- and multi-factor analysis of variance and Dwass-Steel-Critchlow-Fligner pairwise comparisons were used for data analysis.

**Results:**

Our research demonstrates that the sibling position of a mother as a sole factor does not have any statistically significant association with her psychological well-being, with the exception of depressive tendencies, which for mothers with siblings who are younger by five or more years are stronger than for mothers with siblings who are younger by less than five years (N=356, W=3.9, p=0.047).

For mothers without childcare experience, the optimism index does not change significantly in relation to their sibling position. For mothers with childcare experience, depending on their sibling position, the level of optimism decreases (starting from mothers without siblings with the highest level of optimism, followed by mothers with older siblings, and then by mothers with younger siblings) (N=469, p=0.3).

Mothers without siblings or with older siblings are associated with a lower level of anxiety as the volume of assistance with their household chores increases, while for mothers with younger siblings, in the case of a large amount of help with household chores, there is a notable increase in trait anxiety (when compared with the scenarios of a lack of help or an average amount of help) (N=392, p=0.031).

No statistically significant associations with maternal burnout symptoms were discovered.

**Conclusions:**

The sibling position of older sister is associated with a certain amount of deterioration in psychological well-being in terms of depression, anxiety and optimism levels. Further research on extensive samples of mothers who are older siblings is advisable.

**Disclosure of Interest:**

None Declared

